# Recent Progress in Cathode Materials for Sodium-Metal Halide Batteries

**DOI:** 10.3390/ma14123260

**Published:** 2021-06-12

**Authors:** Xiaowen Zhan, Minyuan M. Li, J. Mark Weller, Vincent L. Sprenkle, Guosheng Li

**Affiliations:** 1 College of Chemistry & Chemical Engineering, Anhui University, Hefei 230601, China; xiaowen.zhan@ahu.edu.cn; 2 Battery Materials and Systems Group, Pacific Northwest National Laboratory, Richland, WA 99352, USA; minyuan.li@pnnl.gov (M.M.L.); mark.weller@pnnl.gov (J.M.W.); vincent.sprenkle@pnnl.gov (V.L.S.)

**Keywords:** sodium metal-halide battery, intermediate temperature, low-cost cathode, ZEBRA battery, energy storage

## Abstract

Transitioning from fossil fuels to renewable energy sources is a critical goal to address greenhouse gas emissions and climate change. Major improvements have made wind and solar power increasingly cost-competitive with fossil fuels. However, the inherent intermittency of renewable power sources motivates pairing these resources with energy storage. Electrochemical energy storage in batteries is widely used in many fields and increasingly for grid-level storage, but current battery technologies still fall short of performance, safety, and cost. This review focuses on sodium metal halide (Na-MH) batteries, such as the well-known Na-NiCl_2_ battery, as a promising solution to safe and economical grid-level energy storage. Important features of conventional Na-MH batteries are discussed, and recent literature on the development of intermediate-temperature, low-cost cathodes for Na-MH batteries is highlighted. By employing lower cost metal halides (e.g., FeCl_2_, and ZnCl_2_, etc.) in the cathode and operating at lower temperatures (e.g., 190 °C vs. 280 °C), new Na-MH batteries have the potential to offer comparable performance at much lower overall costs, providing an exciting alternative technology to enable widespread adoption of renewables-plus-storage for the grid.

## 1. Introduction

Mass deployment of renewable energy is crucial to achieve ambitious net-zero power generation in the 21st century. While renewables such as wind and solar are increasingly cost-competitive with conventional fossil-fuel-based power generation, intermittency precludes straightforward incorporation into the current power grid and can even potentially destabilize it with supply/demand mismatch (e.g., >20% in supply), leading to negative pricing and wasted power [1]. These factors motivate large-scale, and potentially long-duration, energy storage coupled with renewable electricity production, which can improve revenue generation by storing electricity during low-demand periods and delivering it to the grid during high-demand periods [2]. Of the various energy storage technologies available, electrochemical energy storage is the most generally deployable, not requiring specific geography like pumped hydro or compressed air energy storage and has a wide range of chemistries allowing tuning of energy or power density for the desired application [1,3].

Unfortunately, most electrochemical energy storage technologies coupled to a renewable power source are not economically competitive with fossil fuels at present. A recent technoeconomic analysis indicates that energy storage capacity with a cost less than $20 per kilowatt-hour (kWh) is required for meeting 100% of demand with renewables [3]. If demand only needs to be met 95% of the time, a more achievable but still very low cost of $150 per kWh for energy storage capacity is feasible [3]. Various electrochemical energy storage technologies are being investigated for grid-level storage, including common low-temperature batteries such as lead-acid batteries, redox-flow batteries (RFB), and lithium-ion batteries (LIB) [1,4,5]. Others include high-temperature batteries utilizing molten Li anodes such as Li-S or Li metal-halide (Li-MH) batteries [6,7,8,9] or molten Na anodes such as Na-S or Na-MH (sometimes called ZEBRA [10,11]) batteries [1,4,5,12]. Among these battery technologies, conventional LIBs have been widely used in various consumer electronics and electrical vehicle applications owing to their high energy density, and they heavily dominate current grid scale demonstrations worldwide. However, LIBs rely on scarce raw materials and have major safety concerns associated with the reactivity and flammability of the organic electrolyte, which call into question the viability of large-scale deployment. Thus, identifying new battery technologies that maximize energy density, rely on inexpensive, Earth-abundant materials, and offer high battery safety, is crucial to enable substantial grid decarbonization and widespread use of renewables. Therefore, targeted development of electrochemical energy storage technologies with both performance and economics in view is needed.

**Figure 1:**
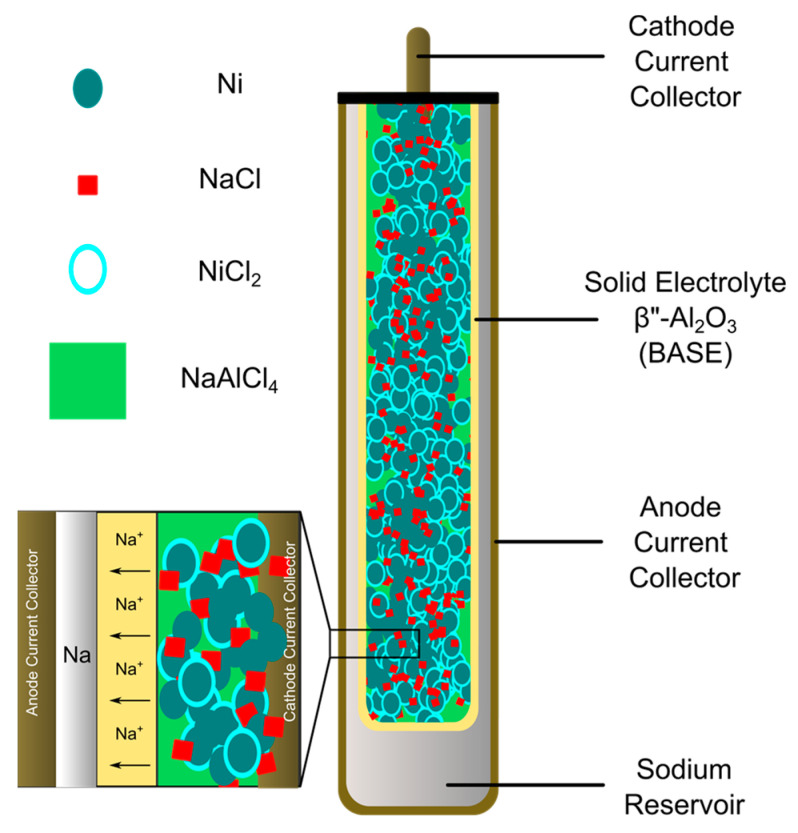
Schematic illustration of a Na-NiCl_2_ battery with a tubular β″-Al_2_O_3_ solid electrolyte (yellow). During operation, molten NaAlCl_4_ acts as the secondary electrolyte in the cathode to conduct sodium ions between the active materials and the solid electrolyte. Particles of Ni form a conductive network radiating from the cathode current collector. A surface coating of NiCl_2_ develops on the Ni particles, as sodium ions migrate across the solid electrolyte to the anode during charging.

Na-based batteries are of particular interest due to relatively high cell voltages (~2 and 2.58 V vs. Na/Na^+^ for Na-S and Na-NiCl_2_, respectively), high theoretical specific energy densities (~790 W h kg^−1^), and the abundance of Na and other components used in the batteries [1,12,13,14]. Both Na-S and Na-MH batteries most commonly use a tubular β″-Al_2_O_3_ solid-electrolyte (BASE) separator to separate molten Na from the cathode material (Figure 1). Na-S batteries use molten S /Na_2_S_x_ as the cathode, whereas Na-MH batteries use a metal halide cathode and NaAlCl_4_ as a secondary electrolyte [15,16]. In both cases, high temperatures (265 C for Na-NiCl_2_, and 350 C for Na-S) are required to promote high ionic conductivity through BASE and enable redox reactions, such as melting sulfur or polysulfides at the cathode and wetting of metallic sodium at the anode. While both types of Na batteries are of technological interest, Na-MH batteries such as Na-NiCl_2_ batteries are particularly promising due to their inherent safety in the case of cell failure relative to Na-S batteries [10,11,12,15,16], the less corrosive nature of the active materials compared to S and polysulfides [12], and the ability to assemble cells in the discharged state [10,11,15,16].

Despite great interest in high-temperature Na batteries, both Na-S and Na-MH batteries are hindered from widespread adoption in grid-level energy storage. Current variations of these batteries using a tubular BASE electrolyte require complex ceramic fabrication process [10,11,15,16]. Further, the materials of construction for containment of the electrode materials, current collectors, and sealing tend to be expensive due to the corrosive nature of the cell components, particularly reactive S/Na_2_S_x_ and NaAlCl_4_ used in Na-S and Na-MH batteries, respectively [1,3,13]. Materials for hermetic sealing of the cell must not only resist corrosion, but also be tolerant of high continuous operating temperatures, which imposes further costs on cell sealing components (glass seals and thermal compression bonding, etc.) and fabrication/processing [4,13,17].

While Na-MH batteries using Ni are promising, the high cost of Ni is a hindrance for further developing cost-effective battery systems. Some researchers have focused on reducing the amount of Ni required for Na-NiCl_2_ batteries by using ‘Ni-less’ batteries at low temperatures [18], as well as using carbon-Ni composites such as Ni-coated graphite [17] or carbon nanowires [19]. Further, cathode degradation due to Ni particle coarsening can severely limit the cycle life of Na-NiCl_2_ batteries [20]. This fact has prompted some researchers to use more complex cathode materials such as Ni nanowires, which can improve cycle life but can be expected to be more costly than conventional Ni powder [21]. Recently, Li et al. demonstrated that cathode degradation in Na-NiCl_2_ batteries can be easily mitigated by operating at a lower temperature (i.e., 190 °C vs. 280 °C), without requiring complex forms of Ni in the cathode [20,22]. In addition to improved cycle life, lower operating temperatures can improve resistance to hot corrosion [4] of cell components as well as enable less expensive materials of construction such as polymer seals instead of glass seals that can operate at lower temperatures [23]. On the other hand, many cost-related challenges with Ni-based Na-MH batteries can be effectively mitigated by replacing Ni with less expensive metals such as Zn [24,25] or Fe [26,27].

Finally, new cell designs based on simple planar geometry along with inexpensive surface treatments [28] effectively mitigate the problem of poor Na-wetting on the solid electrolyte at low temperatures, and also make scalable fabrication of the BASE electrolyte easier, requiring e.g., a simple tape-casting process [20] rather than complex processing of tubular electrolytes [10,11,12,13]. With these major challenges solved, intermediate-temperature, low-cost Na-MH batteries provide new opportunities for scalable, inexpensive, grid-level energy storage to address the urgent need for increased utilization of renewables and their effective incorporation. This review aims to highlight some of the recent work on intermediate-temperature Na-MH battery chemistries, focusing on Ni, as well as low-cost Fe-, Cu-, and Zn-based cathode chemistries that will be crucial to reach the low-cost targets to unlock economical and scalable electrochemical energy storage.

## 2. Ni-Based Cathode Materials for Na-MH Battery Systems

### 2.1. Basic Working Principle

As far as 40 years ago, safety issues in developing high-temperature Na-S batteries prompted active investigation into new cathode materials. While similar corrosion problems persisted, a secondary cell with SCl_4_ cathode in molten NaAlCl_4_ showed attractive energy density and higher battery safety [29]. In 1986, Coetzer et al. reported ZEBRA batteries with transition metal halides as the cathodes in a NaAlCl_4_ secondary electrolyte (m.p. 157 °C), which transported Na^+^ between the active materials and solid-state electrolytes [30]. The solid metal halides were more stable and less corrosive at high temperatures, resolving multiple problems in the Na-S system. Among all metal halides (NiCl_2_, FeCl_2_, ZnCl_2_, CuCl_2_, etc.) compatible with the ZEBRA battery concept, the Na-NiCl_2_ battery has been most widely studied. It was a promising candidate for large-scale energy storage application, with the overall redox reaction below with *E*_0_ = 2.58 V at 300 °C (Equation (1)) [30,31].

2NaCl + Ni (discharged state) ⇄ 2Na + NiCl_2_ (charged state), (1)

To circumvent the handling of anhydrous nickel chloride and highly reactive sodium associated with the charged state, a typical Na-NiCl_2_ battery is usually assembled in the discharged state with nickel powder, sodium chloride, sodium tetrachloroaluminate, and a few additives [10,11]. During charging, Na^+^ ions diffuse through the β”-Al_2_O_3_ solid electrolyte (BASE) and are reduced at the anode side as sodium metal. On the cathode side, oxidized NiCl_2_ coats the Ni surface. During discharging, Na^+^ ions shuttle back through the BASE electrolyte and form NaCl with Cl^−^ ions from NiCl_2_ concurrent with reduction of NiCl_2_ to Ni.

### 2.2. Electrochemical Mechanism and Additives

To take advantage of the high cell voltage, superior energy efficiency, simple assembly, and good cycle life of a Na-NiCl_2_ battery, research efforts have explored the electrochemical mechanism of Na-NiCl_2_ cells in depth and identified factors limiting the cycling performance, including passivation from nickel chloride [22,32], dissolution loss of nickel in the NaAlCl_4_ melt [32], and capacity decline due to Ni/NaCl particle growth. In response, various chemical additives were introduced.

Bones et al. witnessed severe early capacity loss at 250 °C (~50% in <10 cycles), which related to the growth of the nickel grains [33]. At the same time, they found that sulfur additive in NaAlCl_4_ melt could develop an active, metallic phase with high surface area on the cathode. As a result, the long-term cyclability improved, retaining >75% of capacity over 2000 cycles with a negligible increase in the cell resistance [33]. Ratnakumar et al. verified the effect of sulfur additives on limiting Ni growth during cycling and determined the optimal amount between 0.5 and 1.0 wt.% [34]. Prakash et al. identified the nickel chloride layer formation during charging as another factor in the nickel cathode degradation [35]. Although the NiCl_2_ layer initially increased the area capacity of a nonporous nickel electrode by decreasing the impedance between 200 °C and 225 °C, the highly resistive surface layer ultimately led to an accumulation of unutilized NaCl in the chloroaluminate melt and limited the areal capacity at higher temperatures.

In addition to sulfur, researchers explored other additives including NaI/NaBr, Al, FeS, and Ni_3_S_2_. By doping the NaAlCl_4_ melt with NaI or NaBr, NiBr_2_ or NiI_2_ with larger lattice parameters than NiCl_2_ formed in the surface layer. Those surface phases could offer faster mass transport and in turn improve the nickel utilization [32].

**Figure 2:**
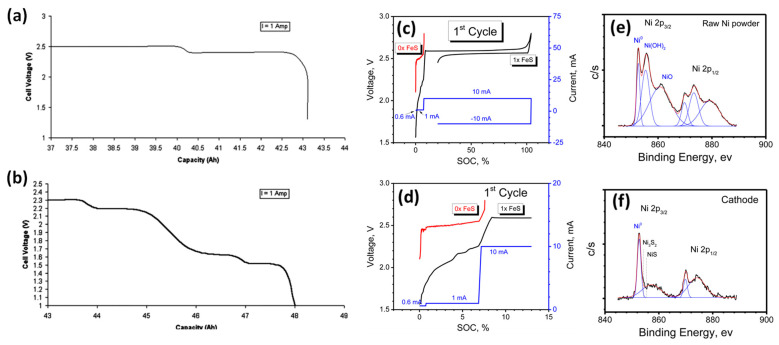
Voltage profiles of (**a**) first-generation and (**b**) second-generation Na-NiCl_2_ cells at the end of discharge. Al and FeS powders were incorporated into the cathode of only the second-generation cell (adapted with the permission from Elsevier). (**c**) Voltage profiles (first cycle) for batteries with and without FeS. (**d**) Expanded view of panel **c**. High-resolution XPS Ni2p spectra for (**e**) raw Ni powder and (**f**) cathode material retrieved from cells charged up to the second step (1 mAh, 10 h, see panel **c**, adapted with the permission from Elsevier).

Al participated in the electrochemical reaction at around 1.58 V (Figure 2a,b) and alleviated the sudden polarization at the end-of-discharge by the following reaction, (Equation (2))

4NaCl + Al ⇄ 3Na + NaAlCl_4_, (2)

Furthermore, the dissolution of Al via Equation (2) left fine pores inside the cathode, which enhanced high-rate performance of the Na-NiCl_2_ battery [11].

FeS showed signature intermediate plateaus in the voltage profile (Figure 2a,b) and could stabilize the nickel grain size similar to elemental sulfur additives [11]. Li et al. systematically investigated the role of FeS in initial activation and subsequent degradation in Na-NiCl_2_ cells. Figure 2c,d shows the first-cycle charge-discharge curves of cells with no FeS (0X) and 1 mol% FeS (1X) loadings. The voltage of a cell without FeS quickly rose to 2.8 V at 10 mA, with no observed plateau at ~2.6 V corresponding to Ni/Ni^2+^. X-ray photoelectron spectroscopy (XPS) analysis on raw Ni powder indicated a passivating surface layer (oxides and hydroxides), which not only increased the ohmic resistance but also decreased the electrochemically active surface area (Figure 2e,f). The addition of FeS largely removed the passivation layer on Ni powder during the low-current charging period, indicated by the shrinking peaks of Ni^2+^ species in XPS. The formation of polysulfide species likely accounted for the surface activation. An optimal level of FeS was necessary to remove surface passivation completely while not inducing Ni particle growth that would later hinder cell performance [36].

**Figure 3:**
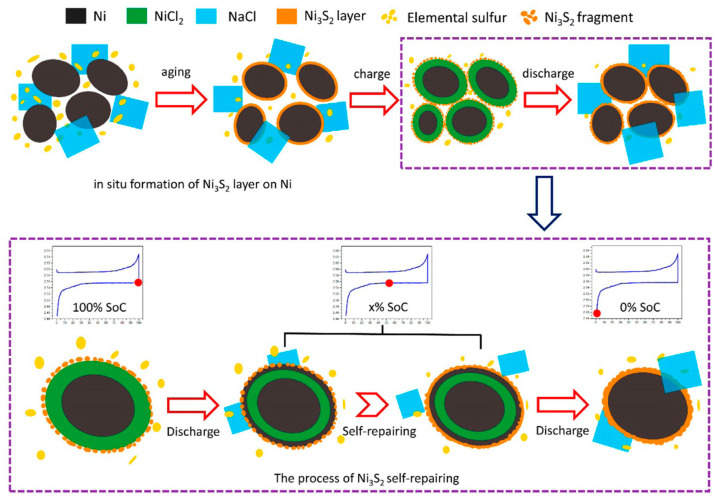
Schematic illustration of the Ni_3_S_2_ layer self-repairing process and the proposed mechanism of sulfur additive via preventing Ni particle growth (adapted with the permission from ACS).

Recently, Ao et al. [37,38] showed that the surface modification of Ni powder by Ni_3_S_2_, from either S or Ni_3_S_2_, can mitigate Ni particle aggregation and promote reversibility. The proposed self-repairing process and mitigation to Ni particle growth relied on in situ Ni_3_S_2_ surface layer formation, schematically illustrated in Figure 3. Although more added sulfur led to the longer sustained self-repairing in the cathode, the layer thickness in excess would degrade cycle performance, owing to the poor electrical conductivity of sulfur and Ni_3_S_2_ [38].

### 2.3. Effects of Operating Temperature and Electrode Morphology

The operating temperature and electrode morphology were two key aspects dictating the interfacial solid–solid conversion, an interplay of thermodynamic and kinetic forces behind performance optimization. Early study by Hosseinifar et al. correlated the performance and aging of ZEBRA cells with operating temperatures of 260 °C and 350 °C [39]. At 350 °C, a combination of high temperature, accelerated NiCl_2_ dissolution in molten NaAlCl_4_, and the formation of the Ni_3_S_2_ phase enabled nickel grain growth, which resulted in a significant capacity loss. Lu et al. constructed a planar-type Na-NiCl_2_ cell that could be cycled at C/3 at a low temperature of 175 °C [40]. Despite the mitigated particle growth, high cell resistance and thus large cell polarization appeared, arising from either slow Na^+^ transport within the NaAlCl_4_ melt or poor wettability of sodium on the BASE at lower operating temperatures.

**Figure 4:**
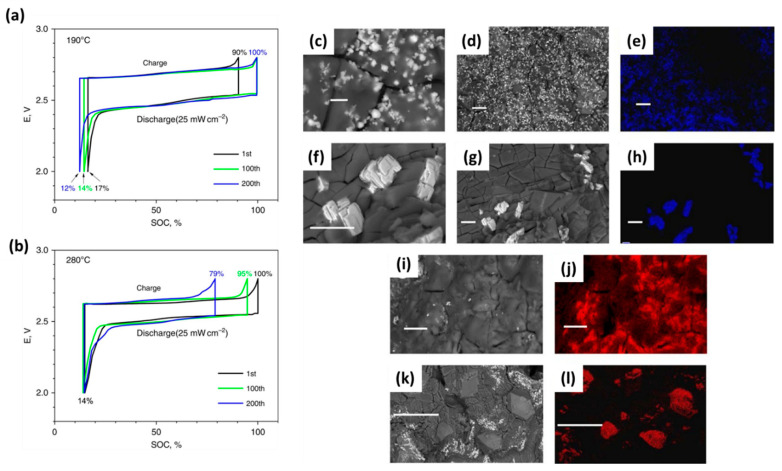
Voltage profiles for planar Na-NiCl_2_ batteries operated at (**a**) 190 °C and (**b**) 280 °C. Cells were cycled with constant-current charge (7 mA/cm^2^, ~C/7) and constant-power discharge (25 mW/cm^2^, 10 mA/cm^2^, ~C/5). Cathode materials were retrieved from cells operated for 200 cycles at 25 mW/cm^2^ and imaged via SEM with EDX mapping analysis (**c**–**l**). Ni particles are shown for cells operated at 190 °C (**c**,**d**) with corresponding EDX mapping of Ni (**e**), and then at 280 °C (**f**,**g**) with corresponding mapping of Ni (**h**). Below, NaCl particles are shown for cells operated at 190 °C (**i**) with corresponding EDX mapping of Na (**j**), and then at 280 °C (**k**) with corresponding EDX mapping of Na (**l**). Scale bars are 2 m for (**c**), 10 µm for (**d**–**j**), 100 µm for (**k**,**l**; adapted with the permission from Springer Nature).

Later, Li et al. demonstrated that a planar Na-NiCl_2_ battery operating at an intermediate temperature of 190 °C with ultra-high energy density (Figure 4) [20]. Discharged at C/5, the cell showed a capacity increase for the first 100 cycles before stabilizing at 137 mAh/g. In contrast, the cell cycled at 280 °C, despite a high initial capacity, exhibited major capacity fading after 100 cycles. Furthermore, the intermediate-temperature cell delivered an impressive energy density of 350 Wh/kg over 1000 cycles with negligible capacity decay. The higher initial capacity observed at 280 °C than at 190 °C was ascribed to the better sodium wetting on the BASE. From the voltage profiles vs. state of charge (SOC) (Figure 4a,b), the cell operated at 280 °C had a stable SOC at end of discharge (SOC_EOD_). However, its SOC at end of charge (SOC_EOC_) dropped rapidly over 200 cycles, indicating that cathode degradation was mainly responsible for capacity fade. This assertion was further supported by morphology analysis using scanning electron microscopy (SEM) with energy dispersive X-ray (EDX) spectroscopy, where the particle growth of both NaCl and Ni at 280 °C was much faster than at 190 °C (Figure 4c,d). Conceptually, lower operating temperatures would not only decrease the solubilities of cathode materials (NaCl and NiCl_2_) but also the diffusion coefficients of the corresponding ions, due to an increased viscosity of the melt. This combined effect hampers diffusion-influenced growth [41]. As a result, Ostwald ripening, previously identified as the underlying particle growth mechanism at 280 °C [22], is significantly suppressed at lower operating temperatures to maintain metal grain size and prolong cell life [20].

Since Na-MH batteries rely on solid–solid conversion chemistry, a large, active cathode interface between NaCl and Ni is important to achieve good electrochemical performance. Prakash et al. investigated three different cathode morphologies (a nonporous nickel substrate, a nickel felt, and a porous sintered nickel electrode) and achieved the best cell performance with the porous sintered electrode. They demonstrated that both surface area and the pore accessibility were critical to performance optimization [42]. Kim et al. compared the electrochemical performance of Na-NiCl_2_ cells using Ni powders with different initial particle sizes (0.5, 6, and 30 µm), showing that smaller particle size (i.e., larger surface area) allowed higher initial charge capacities and more complete utilization of the NaCl [43]. However, capacity fade occurred quickly in cells initially assembled in the discharged state with smaller Ni particles, apparently resulting from particles becoming disconnected from the electron conduction framework due to volume expansion from the formation of surface NiCl_2_ on charging followed by volume contraction on the reduction back to Ni [43]. This problem was mitigated by using ‘reticular’ Ni to maintain the large surface area of the active material while ensuring an interconnected electron conducting framework.

Li et al. carried out extensive studies of the cell performance in correlation to morphology evolution of NaCl and Ni particles, by varying Ni/NaCl ratios and cut-off voltages for EOC [22]. A high current density and a low Ni/NaCl ratio favored the Ni particle growth, while a wide cycling capacity window was mainly responsible for the growth of NaCl. More importantly, excess Ni alleviated performance degradation due to particle growth by offering an additional active electrode surface and electron pathways. As a result, the cell with a cathode Ni/NaCl ratio of 1.8 was able to maintain high capacity over extended cycling. To further lower cell cost by cutting expensive Ni, a recent work by Chang et al. reduced the Ni/NaCl ratio to 1.25 without losing the cell performance at 190 °C [18]. Among many Ni/NaCl ratios, a Ni/NaCl ratio of 1.25 delivered a cell with the highest specific energy density of 405 Wh/kg, with almost no capacity fading over 300 cycles. Morphology analysis showed no significant particle growth after 300 cycles for cells with Ni/NaCl ≥ 1.25. Although the cell did not have the highest capacity at high currents due to low Ni content, it was the most cost-effective with a 30% lower cost of Ni.

Clearly, the microstructure of the cathode material, which enables electrical connection to small, high-surface-area particles, has a significant influence on electrochemical performance over extended cycling and is therefore crucial to optimize. In another study, a core-shell microarchitecture of nickel-coated graphite reduced the Ni loading by 40%. The cell operating at 190 °C showed high initial energy density (133 Wh/kg at ~C/4) and excellent capacity retention (94% over 150 cycles). The decent electrochemical performance was ascribed to the fact that the graphite core successfully retained active surface and structural integrity for fast electron transport [17].

**Figure 5:**
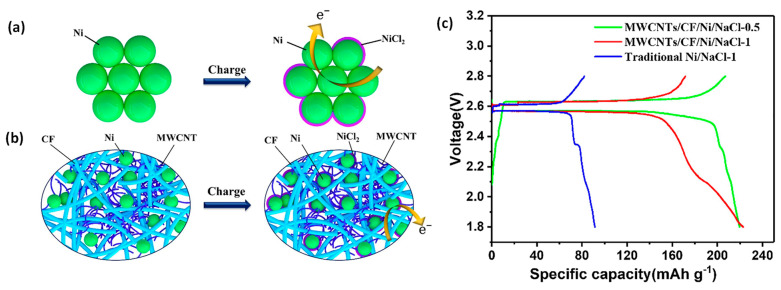
Schematic illustration of the charging process for (**a**) traditional Ni particles and (**b**) the MWCNTs/CF/Ni/NaCl cathode. (**c**) Typical voltage profiles of the traditional Ni/NaCl and MWCNTs/CF/Ni/NaCl cathodes with Ni/NaCl molar ratio of 0.5 or 1 (adapted with the permission from Elsevier).

The incorporation of carbon-based current collectors into the Ni cathode was further developed by Wen’s group through nano engineering [19,21,44]. This approach was effective in providing a continuous electron-conducting network and mitigating particle growth of NaCl and Ni. For example, a free-standing Ni-less cathode with Ni/NaCl particles uniformly distributed in the three-dimensional (3D) conductive matrix constructed from carbon fiber (CF) and multiwalled carbon nanotubes (MWCNTs) is shown in Figure 5a,b [44]. The 3D hierarchical structure via a low-cost vacuum infiltration method (Figure 5a) offered sufficient void space to accommodate the NaCl particle growth and effectively enhance the electronic conductivity of the composite cathode. As a result, at a Ni/NaCl ratio of 1, the MWCNTs/CF/Ni/NaCl-1 cathode achieved an initial reversible capacity of 172 mAh/g, up to 96.5% of its theoretical capacity. In stark contrast, the traditional Ni/NaCl cathode only delivered around 40% of its theoretical capacity in the first conditioning cycle. At a lower ratio of Ni/NaCl of 0.5, the MWCNTs/CF/Ni/NaCl-1 cathode exhibited a capacity of 215 mAh/g, which further confirmed the excellent electronic conductivity within the 3D porous network (Figure 5c). Recently, Gao et al. reported another high-rate and long-life intermediate-temperature Na-NiCl_2_ battery enabled by a dual-functional Ni-carbon composite nanofiber (NCCN) network. The NCCNs could not only offer a continuous conductivity network but also limit the growth of Ni and NaCl grains [19]. The porosity of the cathode, which was controlled primarily by the grain size of the cathode metal, can now be tuned with precise structural engineering on a nanoscale.

### 2.4. Cathode Reaction Kinetics with Respect to Halides

Reducing the operating temperature of Na-NiCl_2_ battery can provide higher long-term cyclability and the adoption of inexpensive polymer materials for cell sealing [20]. However, the reduction in operating temperature results in inferior rate performance. Specifically, the Na-ion transport within the BASE, within the secondary electrolyte, and through the BASE/Na interface, is slower at lower temperatures. More significantly, the cathode reaction processes (such as the formation of NiCl_2_ and dissolution of NaCl) critical to the battery rate performance are also more sluggish. If Na-MH batteries are to become commercially competitive with increased market penetration, the reaction kinetics at lower temperatures require significant improvement [4].

**Figure 6:**
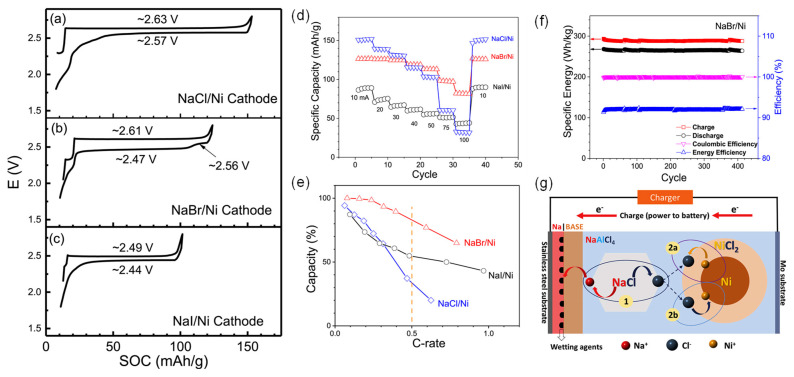
(**a**–**c**) Typical voltage profiles during the conditioning cycles for the (**a**) NaCl/Ni, (**b**) NaBr/Ni, and (**c**) NaI/Ni cathodes. The current density is 3.3 mA/cm^2^ for both charge and discharge, equivalent to ~C/16, ~C/13, and ~C/10 for NaCl/Ni, NaBr/Ni, and NaI/Ni, respectively, and (**d**) plots of the corresponding rate performance in terms of discharge capacity. (**e**) Capacity retention vs. C-rate for NaCl/Ni, NaBr/Ni, and NaI/Ni cells. (**f**) Long-term performance of (**c**) NaBr/Ni cell cycled at a current density of 10 mA/cm^2^. (**g**) Schematic view of reaction mechanisms for a Na-NiCl_2_ battery during charging (adapted with the permission from Elsevier).

Zhan et al. recently adopted a strategy of manipulating the halides in the Na-MH battery cathodes [45]. Comparing the typical voltage profiles of NaCl/Ni, NaBr/Ni, and NaI/Ni cathodes shown in Figure 6a–c, NaCl/Ni and NaI/Ni cathodes exhibited a single plateau for charging and the discharging process. On the contrary, the NaBr/Ni cathode showed an extra discharge plateau at ~2.47 V with a larger overpotential, which was a result of phase relaxations among multiple NaBr_x_Cl_1-x_ based on high-resolution synchrotron X-ray diffraction (XRD) and ion chromatography analysis. This cathode showed a significant improvement in rate capacity among the three compositions (Figure 6d,e) and excellent cycling performance over 400 cycles (>100 days) at 30 mA (10 mA/cm^2^) with negligible capacity fading in the example of NaBr/Ni (Figure 6f). Combining inductively coupled plasma-optical emission spectroscopy (ICP-OES), cyclic voltammetry (CV), and SEM characterizations, the observed rate improvement was attributed to faster NaBr dissolution in molten NaAlCl_4_. 

This finding also strongly suggested that the rate-limiting step of the intermediate-temperature Na-MH batteries was the dissolution of sodium salts (e.g., NaCl) rather than metal halide (e.g., NiCl_2_) formation during the charging process (Figure 6g). While the ion diffusion coefficient of NiBr_2_ was the lowest among the three halide salts obtained through cyclic voltammetry, the solubility of NaBr was the highest obtained through ICP-OES. If the rate-limiting step was the formation of NiX_2_ (X = Cl, Br, or I) during the charging process, then the cell would expect passivation by NiBr_2_ deposition at 100% SOC due to a high concentration of standing Br^−^ and low mobility of NiBr_2_. Instead, no significant features appeared in backscatter SEM images. Furthermore, the low-rate sensitivity of the specific discharge capacity implied sufficient supply of standing Br^−^ at all times, cementing NaX salt dissolution as the rate-limiting step.

## 3. Fe-Based Cathode Materials

### 3.1. Basic Working Principle and Mechanism

**Figure 7:**
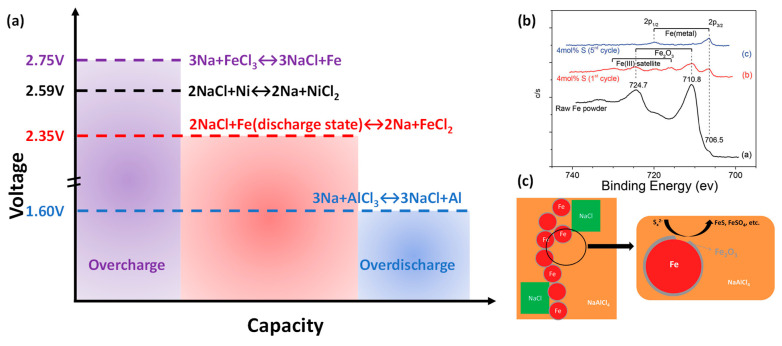
(**a**) Cell reactions in Na-MH battery using an iron chloride cathode. (**b**) XPS Fe2p spectra for raw Fe powder and cycled Fe cathodes (4 mol% S). (**c**) Proposed reaction path for removing oxide layers on Fe particles via polysulfide reaction (**b**,**c**, adapted with the permission from Wiley).

The lower cost of Fe has motivated replacing Ni with Fe as the cathode material during the development of the Na-MH batteries. Examples of solid-state FeCl_2_ cathodes in conjunction with NaAlCl_4_ molten salt as the electrolyte date back to 1980 [30,46,47,48], with an open-circuit voltage of 2.35 V at 250 °C (Figure 7a, red dash line). Like the Na-NiCl_2_ cell, the Na-FeCl_2_ cell was also loaded in the discharged state with Fe and NaCl in the cathode compartment for the ease of assembly. When the cell was overcharged (Figure 7a, purple dash line), the resulting FeCl_3_ that dissolved in NaAlCl_4_ could poison the BASE, leading to a rise in resistance [47]. This undesired side reaction could be mitigated by Ni additives, which offer an overcharge protection by undergoing a reversible cell reaction at 2.59 V (black dash line, Figure 7a) and forming insoluble NiCl_2_. On the other hand, the cell reaction at 1.60 V (blue dash line, Figure 7a) corresponds to the reduction of NaAlCl_4_ to Al and NaCl, serving as a good over-discharge protection. It was also shown to be important to keep the NaAlCl_4_ melt basic (NaCl/AlCl_3_ ≥ 1), ensuring an intimate contact amongst Fe, FeCl_2_, and NaCl in the cathode [46,47].

Unlike a single-stage reaction in Na-NiCl_2_ cell, the partially discharged Na-FeCl_2_ cell contained various intermediate phases with a general formula of Na_8-x_Fe_x_Cl_8_ [48]. At 250 °C, Adendorff et al. found that the electrochemical reaction proceeded in a two-stage manner (Equations (3) and (4)):

6Na + 4FeCl_2_ ⇄ 3Fe + Na_6_FeCl_8_, (3)

2Na + Na_6_FeCl_8_ ⇄ Fe + 8NaCl, (4)

The intermediate Na_6_FeCl_8_ crystalized into a defected rock-salt structure (Fm$\bar{3}$ m) where 1/8 of the octahedral sites were empty. Raising the operating temperature to 350 °C, two other phases, orthorhombic Na_2_FeCl_4_ (Pbam) and trigonal Na_2_Fe_3_Cl_8_ (R$\bar{3}$ m), also formed. Unlike FeCl_3_, the formation of these complex intermediate phases showed neither significant poisoning of the BASE by Fe^2+^ ions, nor structural disintegration of the sintered iron electrode even after hundreds of cycles [48]. Moseley et al. investigated the potential effects of iron incorporation on BASE stability and cell performance in Na/FeCl_2_ cells. Their findings suggested that cells operated under the normal temperature (i.e., 250 °C) with overcharge/over-discharge protections were free from the concerns of iron incorporation into β″ grains [46]. Ratnakumar et al. confirmed the abovementioned stepwise conversion of FeCl_2_ cathode based on cyclic voltammetry results. The potentials of reaction (3) and (4) were determined to be 2.353 V and 2.341 V, respectively, with a separation of 12 mV. Slight asymmetry between the oxidation and reduction peaks was also observed, corresponding to the relative kinetics of individual charge transfer steps and the relative amounts of the intermediate species [49]. Coetzer et al. evaluated the electrochemical behaviors of iron electrodes with NaCl-saturated NaAlCl_4_ in the temperature range 175–400 °C [50]. Remarkably, the results pointed to a predominantly solid-state reaction mechanism near 250 °C, which was further confirmed by the successful operation of a solid-state Na-FeCl_2_ cell with no liquid electrolyte. At high temperatures (300–400 °C), dissolved species such as FeCl_2_ contributed increasingly to the cathode reaction mechanism. Orchard et al. modeled the discharge behavior of the Na-FeCl_2_ cells [51]. Their calculations, where the solubilities of FeCl_2_ and NaCl were disregarded, agreed with the cathode reaction as effectively a solid-state process.

Battery activation and ease of assembly were also important to Na-FeCl_2_ cells. An early approach by Coetzer et al. used pre-sintering of the Fe cathode in a reducing environment to form electron-conducting paths for initial charging of batteries assembled in the discharged state [30,50]. Alternatively, Bones et al. exploited gaseous chlorination of the Fe cathode by Cl_2_ and then assembled the battery with a partially charged Fe/FeCl_2_ cathode and a sodium metal anode [47]. Both approaches introduced complications due to the handling of hazardous/sensitive materials like reduced Fe, FeCl_2_, or metallic sodium. Recently, Li et al. proposed an easy discharged-state assembly of a Na-FeCl_2_ battery utilizing sulfur-based additives [26]. As shown in Figure 7b, the cell with 4 mol.% S addition showed a significant reduction of Fe^3+^ related (Fe_2_O_3_) peaks by XPS, indicating the successful removal of oxide layers on the Fe cathode during the cell conditional cycling. The oxide removal not only freed Fe metal surface for Fe/FeCl_2_ electrochemical conversion (Figure 7c), but also reduced cell resistance to lower overall cell polarization. As a result, the cell using an optimal amount of sulfur additives exhibited excellent cycling performance with no capacity fade and minimum end-of-charge (EOD) voltage degradation up to 100 cycles [26].

### 3.2. Fast-Charging Capability

**Figure 8:**
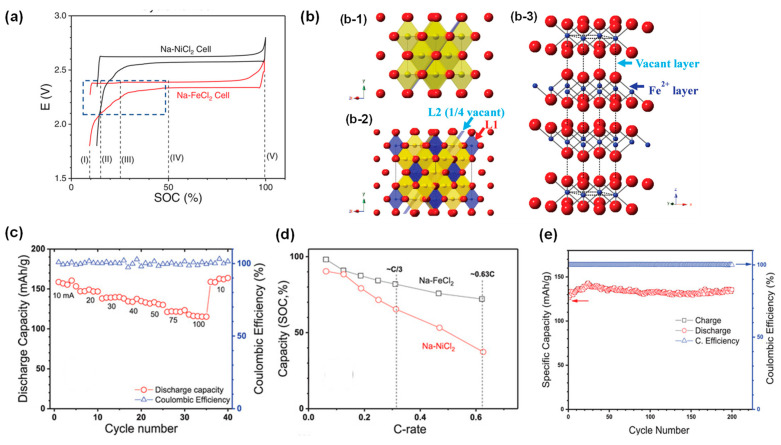
(**a**) Typical voltage vs. SOC plots of Na-NiCl_2_ and Na-FeCl_2_ cells. (**b**) Crystal structures of three key phases, (**b-1**) NaCl, (**b-2**) Na_6_FeCl_8_, and (**b-3**) FeCl_2_, involved in the charging process of a Na-FeCl_2_ battery. The red spheres represent Cl^−^ ions, while yellow and blue octahedra are, respectively, six-coordinated NaCl_6_ and FeCl_6_. The (111) plane is indicated by light blue for NaCl and Na_6_FeCl_8_. (**c**) Rate performance of a Na-FeCl_2_ battery operated at 190 °C. (**d**) The evolution of normalized capacity (SOC, %) as a function of C-rate for Na-FeCl_2_ batteries. The data of Na-NiCl_2_ batteries (red points and line) were included as references. (**e**) Stable long-term cycling performance in terms of specific capacity of Na-FeCl_2_ batteries with 10 wt.% Ni as cathode additive (adapted with the permission from Wiley).

In addition to the low cost and abundance of raw materials (Fe and NaCl), Na-FeCl_2_ batteries have also been demonstrated with fast-charging capability [27]. Following the successful self-activation of a Fe powder cathode enabled by sulfur-based additives, Zhan et al. recently reported a high-rate Na-FeCl_2_ battery operated at 190 °C with voltage profile shown in Figure 8a. NaCl first transformed to Na_6_FeCl_8_ (Equation (5)) then to FeCl_2_ (Equation (6)) during the charging process and overall reaction can be written as Equation (7):

8NaCl + Fe (discharged state) ⇄ 2Na + Na_6_FeCl_8_, 0 25% SOC, (5)

Na_6_FeCl_8_ + 3Fe ⇄ 6Na + 4FeCl_2_ (charged state), 25 100% SOC, (6)

Overall reaction: 2NaCl + Fe ⇄ 2Na + FeCl_2_, (7)

As shown in Figure 8b, NaCl and Na_6_FeCl_8_ shared the same rock-salt structure, despite the latter having a unit cell twice that of the former. Meanwhile, Na_6_FeCl_8_ and FeCl_2_ resemble each other in the arrangement of Cl^−^ ions and alternating Fe^2+^ occupancy, which in part led to fast Na_6_FeCl_8_/FeCl_2_ phase transitions with minimal rearrangement of the atoms. These structural similarities among the three phases facilitated the charging reaction. In addition, CV and Tafel measurements indicated that the Fe/Fe^2+^ redox couple possessed intrinsically faster kinetics than the Ni/Ni^2+^. As a result, the Na-FeCl_2_ battery delivered unprecedented high-rate performance (Figure 8c), with a capacity of 116 mAh/g at an extremely high current of 100 mA (33.3 mA/cm^2^; 0.63C). When expressed as normalized capacity, this Na-FeCl_2_ battery doubled the conventional Na-NiCl_2_ battery at the same rate (Figure 8d). More encouragingly, the optimized Fe/NaCl cathode exhibited excellent cycling stability (Figure 8e) by maintaining a discharge energy density of over 135 mAh/g (295 Wh/kg) for 200 cycles at 10 mA/cm^2^ (≈C/5) [27].

### 3.3. Effect of Electrode Morphology and Fe/Ni-Mixed Cathodes

Like the Na-NiCl_2_ system, controlling the morphology of Fe particles was vital to maintaining a good electron conducting network and ensuring high active material utilization in a Na-FeCl_2_ battery. Ahn et al. prepared Na-(Ni, Fe)Cl_2_ cells with different microstructure designs and investigated the electrochemical behaviors of the composites [52]. Their findings suggested that the conducting metal (higher electrochemical potential, Ni in this case) should have smaller particle sizes than the active metal (Fe in this case) in order to preserve electrical connection in the cathode. With a higher weight ratio of Fe in the composite, the resulting thinner passivating surface NiCl_2_ layer led to better cycling performance [52]. In the aforementioned work conducted by Zhan et al., the pristine Fe cathode (with only Fe and NaCl) showed very early capacity fading. SEM studies revealed considerable pulverization of Fe particles [27]. ICP-OES tests on multiple relevant phases revealed that FeCl_2_ had the highest solubility (0.06 mol%) in NaAlCl_4_, which was twice that of Na_6_FeCl_8_ (0.03 mol%) and 20 times that of NiCl_2_ (0.002 mol%). It was thus proposed during the solid–solid conversion from FeCl_2_ to Na_6_FeCl_8_ and finally to Fe at discharge, that reduction of dissolved species from the liquid electrolyte formed fine Fe particles. These Fe particles, smaller in size with undefined precipitation locations, were more likely to depart from the electron-conduction network and become electrochemically inactive. Consequently, the proportion of active Fe particles gradually decreased with cycling, leading to the observed capacity fading. Adding 10 wt% Ni strengthened the electron-conducting chain (Figure 7c), which successfully inhibited the Fe particle pulverization and enabled stable long-term cycling (Figure 8e) [27].

## 4. Other Transition Metal Halide Cathode Materials

**Figure 9:**
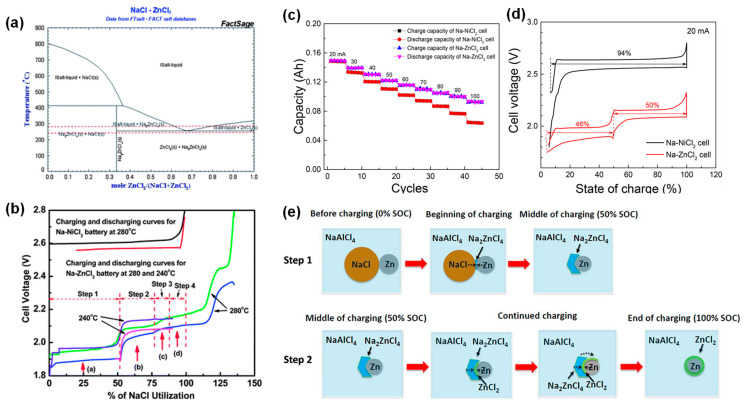
(**a**) Phase diagram between NaCl and ZnCl_2_. (**b**) Initial charge and discharge curves of a Na-ZnCl_2_ battery at 280 °C and 240 °C compared with a Na-NiCl_2_ battery (adapted with the permission from RSC). (**c**) Charge/discharge capacity of Na-ZnCl_2_ and Na-NiCl_2_ cells at different charging/discharging rates. (**d**) Voltage profiles of the two cells at constant currents of 20 mA. (**e**) Cathode reaction mechanisms for Na-ZnCl_2_ battery (adapted with permission from ACS).

In addition to extensive development of cathodes based on ferrous and nickel chlorides, other transition-metals have also been explored. In an early study, Ratnakumar et al. demonstrated that the feasibility of a metal chloride as a cathode material was predictable by qualitatively estimating the solubility in NaAlCl_4_ molten electrolyte through CV [49]. Specifically, the higher oxidation peak current than the corresponding reduction implied either a porous deposit or high solubility of the oxidation product [49]. Based on this criterion, they conducted screening tests for several candidates including manganese, chromium, aluminum, silver, titanium, molybdenum, and cobalt, among which only Co and Mo chlorides displayed suitability at 250 °C with open-circuit voltages of 2.55 V and 2.64 V, respectively [53]. Parthasarathy et al. first reported a high-temperature (350 °C) Na-ZnCl_2_ battery using Zn cathode and a molten NaCl-ZnCl_2_ with over 33 mol% of ZnCl_2_ as the catholyte/cathode. Although the electrochemical study was limited, there was no evidence of ion exchange with BASE, which implied the viability of zinc chloride as a cathode. In 2013, Lu et al. investigated the behavior and performance of a low-cost Na-ZnCl_2_ battery in a planar configuration with BASE [25]. Based on the phase diagram in Figure 9a and the voltage profiles of Na-ZnCl_2_ cells in Figure 9b, the following four reversible stepwise reactions (Equations (8)–(11)) were proposed and validated by phase diagram calculations (NaCl utilizations) and XRD phases analysis.

4NaCl + Zn ⇄ Na_2_ZnCl_4_ + 2Na (E~1.92 V), (8)

Na_2_ZnCl_4_ + Zn ⇄ Salt liquid (ZnCl_2_: 62 mol%) + Na (E~2.07 V), (9)

Salt liquid (lower ZnCl_2_) + Zn ⇄ Salt liquid (higher ZnCl_2_) + Na (E~2.07–2.12 V), (10)

Salt liquid (ZnCl_2_: 78 mol%) + Zn ⇄ ZnCl_2_ + Na (E~2.13 V), (11)

Upon charging, NaCl first reacted with Zn to form Na on the anode side and Na_2_ZnCl_4_ on the cathode side. Once all the NaCl was consumed, Na_2_ZnCl_4_ and Zn reacted to form the NaCl-ZnCl_2_ liquid phase. At the end of charge, the liquid phase reacted with Zn to generate solid ZnCl_2_. The authors further identified the effect of the liquid-phase formation on electrochemical performance by testing cells at 280 °C and 240 °C. At 280 °C, cells revealed stable cyclability with the liquid phase formation during cycling. However, a rapid rise in polarization was observed at 240 °C where only a solid-state reaction occurred. SEM analysis indicated that the growth of Zn and NaCl particles was suppressed at 280 °C, where Zn and NaCl precipitated during discharge [25]. Later, Lu et al. investigated the performance and reaction mechanism of a Na-ZnCl_2_ battery at 190 °C. As predicted by the phase diagram (Figure 9a), two stepwise reversible reactions (Figure 9d) were identified, and the corresponding reaction mechanism was illustrated in Figure 9e. First, NaCl reacted with Zn to produce a ribbon-like Na_2_ZnCl_4_ layer. This layer existed at the NaCl–Zn interface rather than covering the Zn particles’ surfaces, resulting in excellent rate capability (Figure 9c). Subsequently, the Na_2_ZnCl_4_ transformed gradually into ZnCl_2_, which covered the surfaces of Zn particles. The authors suggested that the passivation by ZnCl_2_ during the second step blocked the electron pathway of the NaCl/Zn cathodes and limited the overall rate performance of the battery [24]. More recently, Lee et al. reported an electrochemically activated Na-ZnCl_2_ battery at 260 °C using a carbon matrix in the cathode compartment. By using inexpensive carbon felt to maintain efficient electron percolation in the cathode, the cell realized lower charge transfer resistance and higher capacity, as well as lower materials cost [54].

CuCl_2_ is also an attractive cathode material because of its high theoretical capacity of 400 mAh/g, with equilibrium potentials of 3.40 V for the CuCl_2_/CuCl redox couple and 2.74 V for the CuCl/Cu redox couple in Na-based battery systems [24,55,56]. However, CuCl_2_ is soluble in conventional organic-based liquid electrolytes and high-temperature molten salt electrolytes such as NaAlCl_4_, hindering its practical application. In 2014, Yang et al. proposed an advanced intermediate-temperature Na/CuCl_2_ battery using a NaCl-EMIC-AlCl_3_ catholyte and a 500 mm thick BASE film separator operating at only 150 °C [57]. Unfortunately, the study did not report cyclability or identify the fundamental parameters such as the stability of CuCl_2_. In the search for suitable electrolytes compatible with CuCl_2_, Kim et al. constructed a new ZEBRA-type room temperature Na-CuCl_2_ battery system with SO_2_-based nonflammable inorganic electrolyte. The Na-CuCl_2_ cell showed outstanding battery performance with a high energy density of ~580 Wh/kg based on the cathode material, a more than 97% round-trip efficiency, as well as a superior capacity retention over 1000 cycles. These excellent performances were attributed to the SO_2_-based inorganic electrolyte, which exhibited high ionic conductivity and high chemical/electrochemical compatibility with the CuCl_2_ cathode material [55].

As a final comment, a major driving force behind the intermediate-temperature sodium battery is that sodium is significantly more abundant than lithium, which provides an economic incentive for long-duration and thus low-cycle battery applications. While other metals can participate in the conversion chemistry as shown above, the scale of the upcoming energy storage target prefers an inexpensive cathode metal where the chemistry is well-understood, such as Ni or Fe.

## 5. Conclusions

Replacing fossil fuel-based power generation with widespread deployment of renewable energy sources is a crucial technological goal for reducing greenhouse gas emission. For this to occur, the inherent intermittency of renewables such as wind and solar must be addressed by some form of energy storage. Electrochemical energy storage using batteries is a promising option, with various storage technologies such as LIBs, Na-S batteries, RFB, and also Na-MH batteries. For such technologies to be adopted, they must have desirable performance metrics such as high voltage, high specific energy density, high-power density, and superior safety and reliability while also being inexpensive enough to be economical. Of these options, Na-MH batteries are promising due to their high operating voltage, low cost of materials, good safety in the case of cell failure, and ability to be assembled in the discharged state. However, challenges regarding high operating temperature, high cost of materials of construction, and the high cost of Ni has motivated developing Na-MH batteries with low-cost transition metal cathodes and intermediate-temperature operation.

In this review, Na-NiCl_2_ batteries have been discussed with a particular focus on recent work that addresses some of the challenges of conventional Na-MH batteries, specifically by using lower operating temperatures (e.g., 190 °C rather than >265 °C or higher), identifying methods to reduce the amount of expensive Ni required, employing lower-cost materials for sealing (enabled by lower temperatures), additives that improve otherwise sluggish kinetics at lower operating temperatures, and others.

Moving beyond expensive Ni, other transition metal cathodes such as Fe, Zn, or Cu, are of great interest due to the greater abundance and lower cost of raw materials. Some of these battery chemistries have been discussed in this review, showing comparable performance to widely studied Na-NiCl_2_ batteries but with lower-cost active materials. In some cases, these chemistries even offer superior performance compared to Na-NiCl_2_ cells such as the high rate capability of Na-FeCl_2_ cells or the high theoretical specific capacity of the Na-CuCl_2_ cells discussed herein. While each of these transition metal battery chemistries have some general similarities to the Na-NiCl_2_ system, each also has unique benefits and challenges. Although there have been valuable explorations of these non-Ni cathode systems to date, there is still much room for continued investigation to identify further potential improvements. For example, further reduction in operating temperature (e.g., <120 °C) of Na-MH batteries can expand the potential materials of construction while requiring less thermal energy management and improving safety. Such temperature reductions will require new innovations to improve Na-wettability on the anode side of BASE as well as improvements in catholyte composition to reduce the melting point while maintaining good kinetics and mass transfer on the cathode side. Potentially, interface engineering of the Na-BASE solid interface could enable high-performance solid-sodium Na-MH batteries operating at temperatures below 100 °C. Finally, the demonstration of large area planar Na-MH cells is a crucial step to scaling up this promising battery concept from the lab to the grid scale.

We expect that continued research efforts to enable intermediate-temperature Na-MH batteries with low-cost cathodes will allow battery performance that can surpass current state-of-the-art molten Na batteries while also providing much lower overall energy storage costs. By improving performance and reducing cost, not to mention taking advantage of future economies of scale, intermediate-temperature Na-MH batteries will be a critical component of an economical, renewable power grid.
